# The White Coat Effect Influences Intraocular Pressure Measurements in Dogs: Comparing Tonometry Values Obtained in the Clinic Versus Home

**DOI:** 10.1111/vop.70113

**Published:** 2025-11-16

**Authors:** Ruth Pentlarge Barrow, Travis Strong, Kazuya Oikawa, Gillian J. McLellan, Ellison Bentley

**Affiliations:** ^1^ Department of Surgical Sciences School of Veterinary Medicine Madison Wisconsin (WI) USA; ^2^ McPherson Eye Research Institute Madison Wisconsin (WI) USA; ^3^ Department of Ophthalmology and Visual Sciences, School of Medicine and Public Health University of Wisconsin‐Madison Madison Wisconsin USA

**Keywords:** canine, glaucoma, intraocular pressure, ocular hypertension, tonometry, white coat phenomenon

## Abstract

**Objective:**

To determine the effect of clinic versus home environments on intraocular pressure (IOP) measurements in normotensive dogs.

**Animals:**

Forty client‐owned normotensive dogs.

**Methods:**

In a prospective crossover study, 40 normotensive dogs were included following a complete ophthalmic examination. IOPs were measured in both eyes for each dog using a TonoVet rebound tonometer by a single observer. IOPs were measured in the clinic after the ophthalmic examination, and in the home upon initial entry by the observer (t = 0) and 10 min later (t = 10). Paired t‐tests were performed to compare IOP in the clinic versus home and adjusted *p* < 0.05 followed by the Holm‐Šídák correction were considered significant.

**Results:**

Mean IOP in the clinic (15.7 mmHg ±2.7) was significantly higher than mean IOP in the home at both t = 0 (14.2 mmHg ±2.2; *p* < 0.0001) and t = 10 (12.9 mmHg ±1.9; *p* < 0.0001). Mean IOP in the home at t = 0 (14.2 mmHg ±2.2) was significantly higher than mean IOP in the home at t = 10 (12.9 mmHg ±1.9; *p* = 0.0001). There was no significant effect of testing site sequence on IOP.

**Conclusion:**

IOPs measured by tonometry in the clinic are elevated relative to IOPs obtained in the home, by up to 10 mmHg in one eye in this study. Changes in IOP may be rapid, as seen with the significant reduction in IOP over 10 min of acclimation in the home setting. Veterinarians should consider these findings when interpreting IOP values, especially in dogs that are stressed by the clinic environment.

## 1 Introduction

Glaucoma refers to a group of diseases characterized by degeneration of retinal ganglion cells and the optic nerve, leading to vision loss and blindness. Although glaucoma is multifactorial in terms of etiologies, an increase in intraocular pressure (IOP) secondary to decreased outflow of aqueous humor is still considered the main risk factor for development and progression of glaucoma in veterinary patients [1]. Therefore, lowering IOP via pharmacological or surgical treatment is the current mainstay of glaucoma treatment in animals [2]. As a result, proper assessment and interpretation of IOPs are critical for guiding initiation of treatment and monitoring of treatment response.

IOP may be influenced by factors beyond ocular pathology, including responses to stress. Across multiple species, psychophysiological stressors have been shown to transiently increase IOP [3, 4, 5]. Psychological stressors involving mental arithmetic tasks, as well as mental operation under time and noise pressure, were shown to significantly increase IOP in humans [3, 4]. Relaxation techniques, such as meditation and ocular relaxation exercises, have been associated with significant IOP reductions in humans, suggesting that the state of the patient's stress levels could have an indirect impact on the progression of glaucoma [6, 7]. In nonhuman primates, IOP has been shown to rapidly and significantly increase in response to stressors associated with events leading up to induction of anesthesia [4]. Physical stressors secondary to immobilization techniques in rabbits led to a significant increase in IOPs [5]. However, a clear link between stressors and fluctuations in IOP has not been previously investigated in dogs.

While the effects of stressors on IOP in dogs have not been investigated, “white coat hypertension” has been shown in veterinary species. Blood pressure measurements obtained in the clinic have been shown to overestimate blood pressure relative to those obtained in a dog's home [8]. Retired greyhounds had elevated blood pressures (30 mm Hg (mmHg) higher on average) in the clinic when measured by a veterinarian as compared to blood pressures measured by owners in their home environment [9]. Changes in environment associated with transportation to veterinary clinics, in addition to stressors associated with veterinary examinations, have also been shown to increase systolic blood pressure in cats, confounding in‐clinic blood pressure measurements [10]. It remains unclear how these same factors may confound in‐clinic IOP measurements.

Although the white coat effect classically refers to the tendency of patients to have higher blood pressures in the presence of clinicians, other physiological parameters could be influenced by the white coat effect such as IOP. While the relationship between experimentally induced psychophysiological stressors and IOP has been investigated in the past, there has been no direct investigation evaluating the white coat effect on IOP in veterinary patients to our knowledge. The primary goal of this study was to determine the effect of the clinic versus the home environment on IOPs in normotensive dogs. We hypothesized that IOPs in the clinic would be significantly higher than IOPs in the home.

## 2 Materials and Methods

### 2.1 Animals

A total of 40 dogs were included in this prospective, crossover study. Initially, 47 dogs were enrolled in the study. Each dog had a complete ophthalmic examination performed by a board‐certified ophthalmologist (EB). Any dogs with significant ocular disease were excluded from the study. Six dogs were excluded from the study due to incomplete data. One dog was excluded due to evidence of active chorioretinitis on ophthalmic examination, due to the potential impact of intraocular inflammation on IOP measurements. The use of animals in this study was approved by the Institutional Animal Care and Use Committee at the University of Wisconsin‐Madison (protocol No. V01493‐0‐07‐10). All animal owners provided written informed consent for enrollment in the study, procedures undertaken, and publication of data. All dogs used in this study were owned by students or faculty at the University of Wisconsin‐Madison School of Veterinary Medicine.

### 2.2 Data Collection

Data collected for each dog included age, sex, neuter status, breed, incidental findings on ophthalmic examination, concurrent systemic diseases, past pertinent medical history, current medications, and the time of day that IOPs were measured.

### 2.3 Intraocular Pressure Measurements

IOPs were measured in both eyes for each dog using a rebound tonometer (TonoVet Icare Oy, Finland) without administration of a topical anesthetic [11]. For each measurement, a single TonoVet reading was recorded. Each displayed IOP value represented the TonoVet device's internal measurement of 6 individual IOP measurements, exclusion of the highest and lowest results, and average of the remaining 4 IOP measurements, as per the manufacturer's design. Only readings with none or insignificant deviation indicated by the TonoVet device were included for analysis. All measurements were obtained between 9:30 A.M. and 6:00 P.M. Each dog was measured at approximately the same time of day in the two different locations. Measurements in the clinic and home were obtained within a 1 month period or less for each dog. The mean difference in time of day between clinic and home measurements for the individual dogs was 2.01 h ±1.65 h (median 1.58 h, range of 0 to 5.5 h). Measurements were obtained by a consistent observer (TS) in all dogs in both the clinic and home. IOPs in the clinic were obtained immediately after a full ophthalmic examination. IOPs in the home were obtained immediately after the observer entered the home (t = 0) and 10 min later (t = 10).

### 2.4 Testing Sequence Study Groups

To determine if there was a potential impact of testing site sequence, 18/40 dogs were evaluated in the clinic setting first, and 22/40 dogs were evaluated in the home first. All 40 dogs had IOPs measured at t = 0 in the home. 35/40 dogs also had IOPs measured at t = 10 in the home.

### 2.5 Statistical Analysis

All statistical analyses were performed using Microsoft Excel (Version 365) and GraphPad Prism v10.5 (GraphPad Software Inc.; San Diego, CA, USA). The Pearson correlation coefficient was used to assess the correlation of IOPs between the eyes. The normality of data distribution was assessed using the Shapiro–Wilk test. An unpaired two‐tailed t‐test was used to assess for differences based on testing site sequence (dogs measured in the clinic first versus home first). Paired two‐tailed t‐tests followed by the Holm‐Šídák correction were used to compare the average IOP between the clinic, home at t = 0, and home at t = 10. *p* < 0.05 were considered significant. An a priori power analysis was performed for comparing mean IOP between the clinic and home at t = 10 using a paired two‐tailed t‐test. Detecting a 2 mmHg difference with *α* = 0.05 and 80% power, and assuming a standard deviation of 3 mmHg based on the anticipated variability (−7 mmHg to + 5 mmHg; range 12 mmHg), yielded a minimum required sample size of approximately 18 dogs.

## 3 Results

### 3.1 Animals

Complete signalment data were not available in 5/40 dogs (12.5%). For the dogs with complete signalment data, the mean (standard deviation, SD) age of all dogs was 4.75 (2.79) years (range of 0.15 to 12.93 years) (Table 1). There were 16/35 (45.7%) castrated males, 16/35 (45.7%) spayed females, 1/35 (2.9%) sexually intact males, and 2/35 (5.7%) sexually intact females (Table 1). Intact females were in anestrus during IOP measurements to limit potential hormonal effects on IOPs.

**TABLE 1:** Summary of signalment data for dogs in the 2 study groups (IOP measurements either obtained in the clinic first [*n* = 18] or in the home first [*n* = 22]) and for all dogs in the study (*n* = 40). There was no significant difference in age of dogs between groups (two‐tailed Welch's t‐test; *p* = 0.927).

	Clinic first (*n* = 18)	Home first (*n* = 22)	Total (*n* = 40)
Age (years)			
Mean	4.80	4.71	4.75
Std. Deviation	2.38	3.17	2.79
Median	4.05	4.81	4.3
Minimum	2.37	0.15	0.15
Maximum	10.24	12.93	12.93
Sex			
Female Intact	0	2	2
Female spayed	7	9	16
Male intact	0	1	1
Male neutered	9	7	16
Breed (number of unique breeds)	15	13	23
Most common breeds	Chihuahua (2)	Mix (4), Lab mix (3), Pug (2), Golden Retriever (2)	Mix (5), Lab mix (3), Pug (3), Golden Retriever (3)
Unknown signalment data	2	3	5

A total of 15 dog breeds were represented in this study. The most common breeds were mixed breed dogs (15/35 dogs, 42.9%), Pug (3/35 dogs, 8.6%), Golden Retriever (3/35 dogs, 8.6%), Chihuahua (2/35 dogs, 5.7%), and Labrador Retriever (2/35 dogs, 5.7%) (Table 1). There was equal representation (1/35 dogs, 2.9%) for the following breeds: Dachshund, Standard Poodle, Jack Russell Terrier, Blue Tick Coonhound, Brussels Griffon, Great Pyrenees, Papillon, Cavalier King Charles Spaniel, English Springer Spaniel, and Miniature Schnauzer.

Past medical history was reviewed for each of the 40 dogs included in the study, of which 29 (72.5%) had no past medical history or concurrent diseases. Each of the following conditions was reported in 1 dog (2.5%): historical episode of gastrointestinal signs and elevated liver enzymes (resolved with Clavamox treatment approximately 6 months prior to the study start date), orthopedic issues, anal sacculitis, historical *Anaplasma* positivity, historical pelvic limb amputation, skin fold pyoderma, discoid lupus, grade IV/VI heart murmur, and historical allergies. One dog had reported night vision problems; however, electroretinogram was unremarkable. One dog underwent entropion surgery approximately 6 months prior to inclusion in the study, had a history of atopic dermatitis for which a course of prednisone had been completed greater than 2 months prior, and exhibited persistent conjunctival hyperemia at the time of the study (ophthalmic examination was otherwise unremarkable). A total of 6 dogs (15%) were receiving medications or supplements at the time of the study. Each of the following was administered to 1 dog: Hill's j/d and Dasuquin for joint support, Proin, topical betamethasone, tramadol, fluoxetine, and aspirin.

### 3.2 Ophthalmic Examinations

On ophthalmic examination, incidental findings were noted in 11/40 dogs (27.5%). Six dogs (15%) had incidental cataracts including pulverulent cataracts in 3 dogs (7.5%), punctate axial cataracts in both eyes in 1 dog, punctate anterior cortical cataract in the left eye in 1 dog, and an incipient equatorial cortical cataract in the right eye in 1 dog. Three dogs (7.5%) had mild chorioretinal scarring, including multifocal peripapillary retinal scarring in both eyes in 1 dog, peripheral retinal scarring in both eyes in 1 dog, and retinal scars in both eyes in 1 dog. The chorioretinal scarring was consistent with resolved, inactive disease. These dogs had no evidence of active inflammation or other concurrent ocular disease at the time of examination and were therefore included in the study. Two dogs (5%) had evidence of iris atrophy in both eyes. Individual cases were noted of each of the following: vitreal floaters in the right eye, retinal folds in the right eye, and focal corneal pigmentation in the left eye. Although corneal pigmentation has the potential to affect IOP measurements, the corneal pigmentation noted was focal, small, and peripherally located near the limbus; therefore, this dog was included in the study.

### 3.3 Intraocular Pressures Measurements

A high inter‐eye correlation in IOPs was found between right and left eyes within dogs (*r* > 0.7, *p* < 0.001) across all three study conditions. Thus, IOPs for both eyes were averaged to obtain a single IOP value for each subject for subsequent statistical analyses. There was no significant effect of testing site sequence (home‐first versus clinic‐first measurement) on IOP (*p* = 0.60). Therefore, dogs evaluated in the clinic first and home first were evaluated together for subsequent statistical analyses.

**FIGURE 1:**
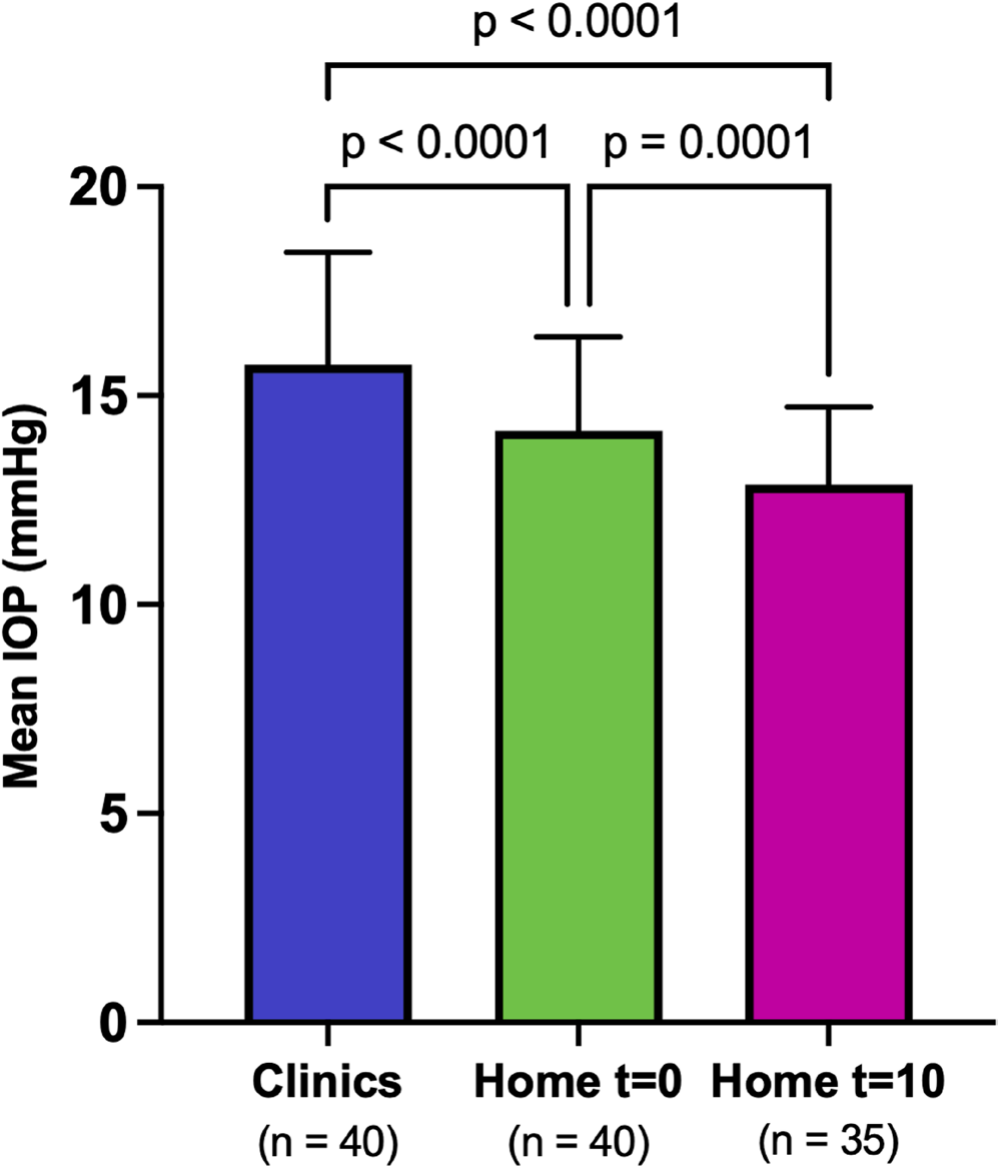
Bar graph showing mean intraocular pressure (IOP) values obtained in the clinic (Clinic), in the home at baseline (Home t = 0), and in the home after 10 min of acclimation to the observer (Home t = 10). Error bars represent standard deviation. P‐values are displayed above the bars comparing the different groups (paired two‐tailed t‐tests followed by Holm‐Šídák correction; *p* < 0.05 was considered significant). Mean IOP was significantly lower when measured in the home than in the clinic setting and was significantly lower at t = 10 than at t = 0 in the home setting.

Mean IOP in the clinic (15.7 mmHg ±2.7) was significantly higher than mean IOP in the home at t = 0 (14.2 mmHg ±2.2; adjusted *p* < 0.0001) and in the home at t = 10 (12.9 mmHg ±1.9; adjusted *p* < 0.0001). Furthermore, mean IOP in the home at t = 0 was significantly higher than mean IOP in the home at t = 10 (adjusted *p* = 0.0001; Figure 1). The mean individual difference in IOP (average of OD and OS) between the clinic and the home at t = 10 was 3.0 mmHg ±2.3 mmHg, with a range of−1.5 mmHg to 7.5 mmHg. The mean individual difference in IOP in the right eyes between the clinic and the home at t = 10 was 2.8 mmHg ±2.6 mmHg, with a range of −3.0 mmHg to 9.0 mmHg. The mean individual difference in IOP in the left eyes between the clinic and the home at t = 10 was 3.2 mmHg ±3.1 mmHg, with a range of −3.0 mmHg to 10.0 mmHg. The largest difference in average IOP (average of OD and OS) between the clinic and home at t = 10 was 7.5 mmHg. Even larger differences were observed when looking at individual eyes, with a maximum difference in IOP of 10 mmHg in one dog between the clinic and home at t = 10.30/35 dogs (86%) had higher IOPs in the clinics than in the home at t = 10.16/35 dogs (46%) had an IOP increase that was greater than 5 mmHg in the clinic relative to home at t = 10 in at least one eye, demonstrating that IOP was significantly labile in some dogs dependent on environment.

## 4 Discussion

This study demonstrated that IOPs measured by rebound tonometry in the clinic setting in a cohort of dogs with normotensive eyes were significantly higher than IOPs measured in the home setting, both at the time of initial observer entry into the home and again 10 min later. These results support our hypothesis that IOP measurements in dogs are higher in the clinic than at home, providing evidence that IOP may be influenced by the white coat effect or other stressors.

Additionally, IOPs measured in the home were significantly higher at initial observer entry compared to 10 min later following acclimation to the observer being in the home setting. This decrease in IOP measurements after acclimation to a change in their environment suggests that environmental stressors play a role in transiently increasing IOP, even in the home setting. Therefore, environmental stressors associated with clinic visits, such as the ophthalmic examination, other animals in the clinic, and restraint techniques, may be contributing to transient increases in IOP. To our knowledge, this is the first study to demonstrate an apparent effect of the white coat effect or environmental stressors on IOP measurements in dogs.

It is noteworthy that some of the dogs demonstrated very labile and generally higher IOP measurements in the clinic setting. The magnitude of this change was variable, with some dogs experiencing only a few mmHg higher IOP in the clinic compared to the home setting. However, some individual eyes exhibited up to 10 mmHg higher IOP in the clinics. Accurate IOP assessment is critical for clinical decision‐making in dogs with, or predisposed to, glaucoma. Therefore, further studies are needed to evaluate the extent to which glaucomatous dogs exhibit IOP elevations in response to stressors.

One important limitation of this study was that behavioral and physiological parameters related to stress in dogs were not assessed and correlated with IOPs. Without assessment of these parameters, it is difficult to confirm a positive correlation between heightened patient stress levels and increased IOPs. However, a significantly higher mean IOP measured in the clinic setting suggests the white coat effect is contributing to higher IOPs in dogs. Future studies assessing stress/behavioral scores and physiological parameters of stress, such as blood pressure and heart rate, in conjunction with IOPs in the home versus clinic environments are needed to further investigate how stress and the white coat effect impact IOPs in dogs. One study showed gabapentin administration in cats reduces stress yet does not lead to a significant decrease in IOP [12]. However, gabapentin administration in dogs has been shown to lead to a decrease in IOP [13]. Further assessment of IOPs in the clinic and home settings in normal dogs versus dogs receiving anxiolytics such as trazodone and gabapentin may provide further support that stressors secondary to the white coat effect are contributing to higher IOPs.

Only obtaining a single measurement in the clinic setting was another limitation of this study, as the data do not establish whether repeated measurements in the clinic may lower subsequent IOPs over time. Demonstrating whether IOPs decrease over time as dogs acclimate to the clinic environment, to a degree similar to the decrease noted as dogs presumably acclimated to the presence of the observer in their home environment, could provide stronger support that stress and the white coat effect are truly leading to an increase in IOP. One study demonstrated a marginally lower IOP in dogs when measurements were repeated during the same visit, suggesting that acclimation to the visit may occur, which follows similar trends seen with blood pressure in humans and veterinary species [14]. The white coat effect on blood pressure in humans has been shown to be transient, with a decrease in blood pressure measurements seen after acclimation to the physician obtaining subsequent blood pressure measurements [15]. In beagles, it has been shown that systemic blood pressure gradually decreases after acclimation to the environment over several weeks [16]. Many patients seen by veterinary ophthalmologists are repeatedly presented to the ophthalmology clinic. It is possible that IOPs obtained in the clinic are not affected by the white coat effect on subsequent visits to the clinic. Conversely, some animals may be increasingly stressed with each visit to the clinic, with the potential of repeated clinic visits leading to progressively higher IOPs with each subsequent visit.

It is important to note that rebound tonometry provides an estimate of IOP. Manometry is the only technique that can directly quantify true pressures within a closed system. Therefore, IOP values discussed in this study should be interpreted as IOP measurements and not necessarily true IOP. Finally, IOP measurements were not externally averaged from multiple separate TonoVet readings, which is another potential limitation in this study. However, each value reflects the device's internal averaging of repeated IOP measurements to minimize potential variability associated with singulate IOP measurements.

This study demonstrates applications for clinical practice including taking steps to decrease patient stress in the clinic, when possible, to obtain IOP measurements that more accurately reflect the IOP exposure in the home environment. By convention, many veterinarians measure Schirmer Tear Tests prior to IOPs, which is commonly considered a stressful test in dogs and could be leading to transiently elevated IOPs based on the findings in this study. Therefore, clinicians may consider measuring IOPs first and then completing the rest of the ophthalmic examination, given the ability to obtain IOP without topical anesthetic with a rebound tonometer. Other considerations to decrease patient stress and reduce the impact of the white coat effect on IOPs include going straight to an examination room upon arrival to the clinic, reducing restraint in dogs that get stressed during examinations, and using anxiolytics such as gabapentin and trazodone for dogs that historically exhibit higher fear, anxiety, and stress scores in the clinic. One study utilized home monitoring of IOPs by training the owners to perform tonometry at home, which could further reduce the white coat effect [17]. In the future with advancing technology, it may even be possible to obtain continuous monitoring of IOP in dogs using devices such as smart soft contact lenses that allow for 24 h monitoring of IOPs [18].

In conclusion, this study demonstrated that IOPs measured in dogs in the clinic were significantly higher than IOPs measured at home. Veterinarians should consider the impact of the white coat effect on IOPs measured in the clinic setting and consider the likelihood that they may be transiently elevated. This may be particularly relevant or evident in stressed dogs. Overall, recognizing the impact of the white coat effect on IOP measurements could lead to more accurate monitoring and enhanced clinical outcomes in canine glaucoma.

## Disclosure

The authors have not used AI to generate any part of the work. This study was approved by the Institutional Animal Care and Use Committee at the University of Wisconsin‐Madison (protocol No. V01493‐0‐07‐10), and all animal owners provided written informed consent for enrollment in the study, procedures undertaken, and publication of data.

## Data Availability

The data that support the findings of this study are openly available in Zenodo at https://zenodo.org/records/16415845.
